# High fat diet improves metabolic flexibility during progressive exercise to exhaustion (VO_2_max testing) and during 5 km running time trials

**DOI:** 10.5114/biolsport.2023.116452

**Published:** 2022-07-19

**Authors:** Philip J. Prins, Timothy D. Noakes, Jeffrey D. Buxton, Gary L. Welton, Amy S. Raabe, Katie E. Scott, Adam D. Atwell, Sarah J. Haley, Noah J. Esbenshade, Jacqueline Abraham

**Affiliations:** 1 Grove City College, Grove City, PA, USA; 2 Department of Medical and Wellness Science, Cape Peninsula University of Technology, Cape Town, South Africa; 3 Youngstown State University, Youngstown, OH, USA

**Keywords:** Crossover point, High fat diet, Low-carbohydrate, High-carbohydrate, Fat oxidation, Carbohydrate oxidation

## Abstract

Recently we reported similar performances in both progressive tests to exhaustion (VO_2_max) and 5km running time trials (5KTT) after consuming low-carbohydrate, high-fat (LCHF) or high-carbohydrate, low-fat (HCLF) diets. Accordingly, we tested the null hypothesis that the metabolic responses during both tests would be similar across diets. In a randomized, counterbalanced, cross-over design, seven male athletes (VO_2_max: 61.9 ± 6.1 mL/kg/min; age: 35.6 ± 8.4 years; height: 178.7 ± 4.1 cm; mass: 68.6 ± 1.6 kg; body fat: 5.0 ± 1.3%) completed six weeks of LCHF (6/69/25% energy carbohydrate/fat/protein) and HCLF (57/28/15% energy carbohydrate/fat/protein) diets, separated by a two-week washout. Substrate utilization and energy expenditure were measured during VO_2_max tests and 5KTTs. The LCHF diet markedly increased fat oxidation and reduced carbohydrate oxidation, with no associated impairment in either the VO_2_max tests or the 5KTTs. Following the LCHF diet, athletes generated 50% or more of their energy requirements from fat at exercise intensities up to 90% VO_2_max and reached the crossover point for substrate utilization at ~85% VO_2_max. In contrast, following the HCLF diet, carbohydrate provided more than 50% of the total energy consumption at all exercise intensities. During the 5KTT, ~56% of energy was derived from fat following the LCHF diet whereas more than 93% of the energy came from carbohydrate following the HCLF diet. This study provides evidence of greater metabolic flexibility following LCHF eating and challenges the popular doctrines of “carbohydrate dependence” for high intensity exercise and the role dietary macronutrients play in human performance.

## INTRODUCTION

It is generally taught that the oxidation of fat by skeletal muscle is unable to provide ATP sufficiently rapidly to sustain exercise of either high or moderate intensity [1]. It has been suggested that the exclusive use of fat cannot sustain metabolic rates during exercise above 50% of the maximum oxygen consumption [2]. Indeed, a popular book [3] by one of the present authors includes the statement that: “At exercise intensities greater than 95% VO_2_max only carbohydrate is burned” so that “The practical point is that at high exercise intensities, carbohydrate, especially muscle glycogen, is used at very high rates and is probably the principal energy fuel” (3, p. 120). This belief originates, at least in part, in a series of iconic Scandinavian studies undertaken in the late 1960s. Adopting the novel percutaneous needle muscle biopsy technique [4], these studies measured the disappearance of glycogen from muscle during both prolonged [5–12] and high intensity [13] exercise. The most frequently cited study [10] reported a linear associational relationship between the pre-exercise muscle glycogen content and the duration of exercise that could be sustained during exercise at high intensity (75% VO_2_max). Many of these studies also reported that the termination of prolonged exercise was associated with near-total muscle glycogen depletion [5, 7, 11, 13].

Two principal conclusions were drawn from these studies: First, that limited glycogen stores is a limiting factor for high intensity exercise [14]. Second, that at low exercise intensities, energy is primarily derived from lipids. However, the fraction of carbohydrate used as an energy source increases with increasing workload, with the result that at 85% to 90% VO_2_max, all energy is derived from carbohydrates [14]. As a result, endurance athletes were advised to eat carbohydrate-rich diets for at least 3 days before competitive events [15].

In 1974, Gollnick [2] provided a theoretical explanation for an essential role for carbohydrate use during exercise at higher intensities: “Part of this may be related to the fact that carbohydrates produce about 5% more energy per liter of oxygen consumed than fats. The ready availability of carbohydrates in muscle may also be a factor. This can be contrasted to the need to transport fats from the blood into the muscle cell and then into the mitochondria. This may well limit the total energy production that can be sustained from the exclusive use of fat. It may also be related to the fact that carbohydrates are necessary to maintain citric acid cycle intermediates at a level needed to support the oxidative capacity of muscle. The most important of these anaplerotic processes is probably the production of oxaloacetate from pyruvate via the pyruvate carboxylase reaction. When the concentration of oxaloacetate in mitochondria is low, entry of acetyl groups to form citrate is reduced. This can retard fatty acid oxidation”. This explanation is known as the “anaplerotic theory” [16] to rationalize why fat oxidation cannot substitute for carbohydrate, especially muscle glycogenolysis, during high intensity exercise. Others propose that glycogen is essential for the optimum calcium release from the sarcoplasmic reticulum so that glycogen depletion may cause fatigue by interfering with skeletal muscle excitation-contraction coupling [17, 18].

Thus, has arisen the belief that exercise of high intensity is “carbohydrate dependent” [3, 19–21] and that “fat-derived ATP production is designed to provide a ‘helper fuel’ during exercise, with a maximum amount of energy at power outputs of ~60–65% VO_2_max [22]” [23]. As a result, “For most events at the Olympics, carbohydrate is the primary fuel for anaerobic and aerobic metabolism” [23] so that “when elite athletes train for and compete in most sporting events, carbohydrate fuels are the predominant and critical substrate for the working muscles, and the availability of carbohydrate [19, 24] rather than fat, wins gold medals” [25].

**FIG. 1:**
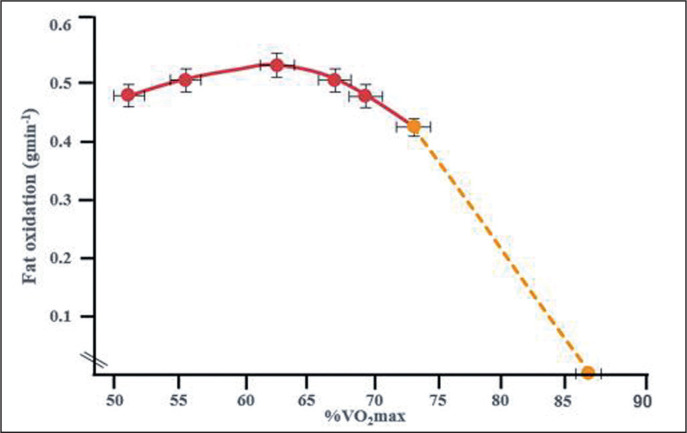
The study of Achten and Jeukendrup [22] found that rates of fat oxidation increase during exercise, reaching a maximum at an exercise intensity of ~65%VO2max before falling to zero at ~85% VO2max in carbohydrate-adapted athletes.

Perhaps the strongest body of evidence supporting this hypothesis is the well-described metabolic response to progressive maximal exercise testing for measurement of maximal oxygen consumption (VO_2_max). Countless studies beginning more than 100 years ago [26] have repeatedly established that the percentage contribution of fat oxidation to energy metabolism falls progressively with increasing exercise intensity. Thus, Achten and Jeukendrup [22] have shown that maximal rates of fat oxidation (0.5 g/min) occur at about 63% VO_2_max with a rapid reduction to 0% at 85% VO_2_max (Figure 1). According to this evidence, carbohydrate becomes the sole energy source during exercise at greater than ~85% VO_2_max. This observation has become known as the “crossover concept” [27–29].

Other studies [30, 31] measuring rates of carbohydrate and fat oxidation during more prolonged exercise confirm that carbohydrate becomes the predominant fuel source used during prolonged exercise at higher exercise intensities of, for example, 65 and 85% VO_2_max [32]; 55 and 75% VO_2_max [33]; 70% VO_2_max [34–37]; and 65% VO_2_max [38]. Even during three hours of lower intensity exercise (55% VO_2_max), carbohydrate provided more than 50% of energy use at the termination of exercise even when no carbohydrate was ingested during exercise [39].

Importantly all these studies were of athletes chronically adapted to high carbohydrate diet so that this “carbohydrate-dependence” could potentially be the result [30, 31] of beginning exercise with high muscle and liver glycogen contents [40, 41]. Indeed, Achten and Jeukendrup [22] reported that the metabolic response during VO_2_max testing showed wide individual variability so that some athletes could cycle up until intensities higher than 85% VO_2_max before any decrease in fat oxidation occurred. They acknowledged that differences in habitual diet might partially explain these different responses. Indeed, many publications now suggest that this “carbohydrate-dependence” may, at least in part, reflect a state of chronic adaptation to a high-carbohydrate low-fat (HCLF) diet that alters quite rapidly on exposure to a low-carbohydrate high-fat (LCHF) diet [24, 42–46].

Recently we reported the results of a dietary randomized controlled trial in which the same subjects performed 5 km time trials following exposure to LCHF or HCLF diets [47]. As the subjects also completed progressive VO_2_max exercise tests to exhaustion when eating either the HCLF or LCHF diets, we realized that a complete data set was available to determine the effects of these two different diets on carbohydrate and fat metabolism during progressive maximal exercise to exhaustion. Since our data showed that performances during both the VO_2_max test and the 5 km time trials were identical on both dietary interventions, we wished to determine whether the metabolic response especially during the VO_2_max test had been influenced by the pre-testing diet. According to the conventional belief that fat oxidation cannot sustain exercise of high intensity, the null hypothesis we evaluated was that the metabolic responses during the VO_2_max tests following either diet must have been identical since the different diets produced identical athletic performances.

## MATERIALS AND METHODS

### Experimental Design

The data presented in this manuscript was collected as part of a larger project [47] examining the effects of LCHF and HCLF diets on running performance, physiological, perceptual, and metabolic adaptations, and change in body composition. Detailed description of general methods and other results can be found in Prins et al. [47].

In brief, participants participated in two 42-day experimental conditions (HCLF or LCHF) in a randomized (www.randomizer.org), counterbalanced, crossover design. The main goal of this study was to determine the effects of the two different diets on carbohydrate and fat metabolism during progressive exercise to exhaustion in the VO_2_max test in highly trained, recreational endurance athletes who underwent 12 weeks of experimental and dietary intervention phases. Participants were instructed to maintain their usual training frequency and log (mode, duration, and intensity of each workout) throughout the study intervention. Additionally, a sequence of tests was performed on day 1, 4, 14, 28, 39, and 42 during each dietary phase. A VO_2_max test was performed at baseline (Day 1) and 6 weeks (Day 39) following the dietary intervention. A 5-km running time trial (5KTT) was performed four times during each dietary intervention (Day 4, 14, 28, and 42). Participants reported to the testing laboratory (Grove City College Exercise Science Human Performance Laboratory) between 6:00 and 9:00 am after an overnight fast (8–12 h).

### Participants

Seven male, competitive recreational runners (age: 35.6 ± 8.4 years; body height: 178.7 ± 4.1 cm; body mass: 68.6 ± 1.6 kg; body fat: 5.0 ± 1.3%; lean body mass: 65.1 ± 1.5 kg; VO_2_max: 61.9 ± 6.1 ml/kg/min; running distance per week: 63.0 ± 27.1 km; running experience: 15.1 ± 7.1 years) volunteered to participate in this study. Inclusion criteria were divided into three objective categories: running performance (< 21’00” 5-km within 3 months of study enrolment; > 32 km of running per week; > 2 years of running experience); age (18–45 years); habitual dietary intake (> 50% total energy intake from carbohydrates). Exclusion criteria included habitually consuming a ketogenic or low-carbohydrate diet (< 20% total energy needs from carbohydrates) or being prescribed lipidor glucose-lowering medications. Participants were prohibited from using any ergogenic aids for one month preceding the study and were asked to refrain from taking any performance enhancing supplement(s) during the course of the study.

Before enrolling in the study, participants were fully informed of any associated risks and discomforts prior to giving their written informed consent to participate. The experimental protocol was approved by the Institutional Review Board of the College prior to implementation. Participants who met the criteria were invited to an in-person consent visit where the protocol and study responsibilities were described in greater detail. After randomization, each participant was assigned to an *ad libitum*, low-carbohydrate/high-fat diet (LCHF**)** or a high-carbohydrate/low-fat diet (HCLF**)** for six-weeks. A two-week, also *ad libitum,* mixed diet (i.e., CHO > 50 g/day and > 20% fat) washout stage separated the two experimental dietary phases.

### Nutrition and Exercise Guidelines

Using direct counselling and prepared educational handouts, a registered dietitian taught and guided each athlete prior to the experimental phase on how to implement the LCHF and HCLF diets at-home. The primary macronutrient targets for LCHF and HCLF were expressed as both a percentage of total daily energy intake and daily gram intake: LCHF: < 50 g/day carbohydrate, 75–80% fat, 15–20% protein; HCLF: 60–65% carbohydrate, 20% fat, 15–20% protein (Table 1).

**TABLE 1:** Dietary composition in the low-fat and low-carbohydrate diet groups over the course of the study.

	Overall Six Week Mean		

Variable	LCHF	HCLF	P Value
**Energy (Kcal/day)**	2947 ± 284	2837 ± 251	0.686
**Carbohydrate (g)**	43 ± 6.0	402 ± 32	0.001
**Protein (g)**	184 ± 28	106 ± 9.0	0.001
**Fat (g)**	226 ± 21	89 ± 14	0.001
**Carbohydrate (%)**	6.0 ± 1.3	56.4 ± 2.6	0.001
**Protein (%)**	25.1 ± 1.5	15.3 ± 1.1	0.001
**Fat (%)**	68.6 ± 2.1	27.8 ± 2.3	0.001
**Cholesterol (mg)**	1199.3 ± 172.1	264.1 ± 29.5	0.001
**Saturated fat (g)**	89.1 ± 13.2	28.7 ± 5.2	0.001
**Monounsaturated fat (g)**	72.8 ± 6.3	25.5 ± 4.5	0.001
**Polyunsaturated fat (g)**	23.4 ± 2.5	14.7 ± 1.9	0.023
**EPA (g)**	0.08 ± 0.04	0.02 ± 0.02	0.019
**DHA (g)**	0.22 ± 0.09	0.04 ± 0.04	0.012
**Fiber (g)**	9.7 ± 1.5	29.6 ± 0.8	0.001
**Sugar (g)**	18.1 ± 2.4	132.4 ± 4.7	0.001

Note: Values are mean ± SD (n = 7). LCHF, low carbohydrate high fat; HCLF, high carbohydrate low fat. Determined from 3 day 24-hour weighed dietary food records including 1 weekend day.

Participants were explicitly instructed to consume the diets until they reached satiety. Consumption of a wide range of foods was encouraged to minimize micronutrient deficiencies. To ensure that mineral status was met, we recommended including an additional 1–2 g/day of iodized table salt to offset the additional loss of sodium associated with a reduction in total carbohydrate intake on the LCHF diet [48]. Weekly energy intake and relative macronutrient distribution was monitored and estimated via 3-day weighed food records, capturing two consecutive weekdays and a weekend day. A digital scale (Ozeri ZK14-S Pronto, San Diego, CA) calibrated to the nearest ± 0.1 g was provided to each athlete prior to experimental phases to improve food tracking accuracy (intended for both dry and cooked items). Dietary macro- and micronutrients were calculated by the same registered dietitian using advanced nutrient software (Nutritionist Pro, Axxya Systems, Redmond, WA). In addition to food records, compliance to the LCHF dietary regimen was monitored by daily, morning capillary blood measurement of beta-hydroxybutyrate (BHB) concentrations (β-hydroxy-butyrate; Precision Xtra, Abbott Diabetes Care Inc., Almeda, CA).

To minimize confounding exercise effects, participants were instructed to select and maintain a constant training intensity and volume that they could adhere to for 14 weeks. Subjects were instructed to record their training habits (mode/duration/intensity) one week prior to commencing the experimental dietary phases. Post-hoc training load analysis revealed no differences in free-living exercise habits throughout the study.

### Testing procedures

#### Progressive Exercise Test to Exhaustion (VO_2_max)

Subjects performed an incremental test to exhaustion on motorized treadmill (Trackmaster TMX425C treadmill, Newton, KS, United States) utilizing the modified Astrand treadmill protocol. Participants began running at a speed between 5–8 mph for 3 min (0% grade). After 3 min of running at 0% grade, the speed was kept constant, and the grade was increased 2.5% every 2 minutes until volitional exhaustion. Heart rate was measured throughout (Polar Electro, Kempele, Finland). The following criteria were used to ensure a physiologically valid VO_2Max_ was attained: (1) a plateau in VO_2_ with increasing exercise intensity (< 150 ml/min or < 2.1 ml/kg/min), (2) a respiratory exchange ratio (RER) of ≥ 1.1, and (3) volitional termination due to exhaustion.

### 5-km running time trial (5KTT)

Participants performed a 5KTT on a motorized treadmill (TMX425C treadmill; Trackmaster, Newton, KS, USA). Before the start of the run, participants completed a 5-min self-paced warm-up run. Participants were instructed to finish the run as fast as possible. The gradient was set at 0.0% grade. Participants were provided with feedback on the distance (at regular 500-m intervals) covered during each TT but were not informed of the overall performance time until completion of the study. During the 5KTT, participants were permitted to adjust their speed whenever they saw fit with the use of control buttons located on the treadmill. The speed indicator and timing devices were concealed from the participant’s view throughout the TT. Therefore, participants regulated their treadmill pace according to their perceived exertion associated with the intensity of the exercise and their subjective feelings of their running capabilities [49].

### Respiratory Gas Analysis

Respiratory gas exchange was recorded using an automated metabolic analyzer system (TrueOne 2400, ParvoMedics, Sandy, UT, United States). Prior to each experimental session, the device was calibrated using procedures according to manufacturer instructions. The breath-by-breath measurements were performed for oxygen uptake (VO_2_), carbon dioxide production (VCO_2_), and respiratory exchange ratio (RER) and was measured continuously throughout trials (VO_2_max and 5-km TT).

The average values for VO_2_ (L/min) and VCO_2_ (L/min) were calculated over the last minute of each 2-min exercise stage in the maximal exercise test, and each minute of the 5KTT. Whole-body rates (g/min) of CHO and fat oxidation, and energy expenditure were calculated using intensity dependent equations that assume negligible protein contribution to energy expenditure [50]. The percentage contribution of CHO and fat to total energy expenditure during exercise was also calculated [27].

### Statistical analysis

Analyses were performed using SPSS Ver. 25 (SPSS, Inc., Chicago, IL, USA). Two-tail *α* significance was set at *p* < 0.05. To address our objective, we analysed main effects and interactions between LCHF and HCLF using a 2 (condition) × 10 (intensity) repeated measures ANOVA to determine differences between LCHF and HCLF during the maximal exercise test for variables with serial measurements e.g., fat oxidation and carbohydrate oxidation (g/min, and % total EE). A 2 (condition) × 5 (distance) repeated measures ANOVA was performed to assess differences between LCHF and HCLF during the 5KTT. All the analysed variables of interest were screened for normality using Shapiro-Wilks’s test. Assumption of sphericity was confirmed using Mauchly’s test; variables that violated sphericity were treated with the Greenhouse-Geiser correction. Bonferroni correction was applied for multiple post-hoc comparisons. All data are presented as mean ± SD. Partial-eta squared (h2p) was used to report effect size with 0.01 considered small, 0.06 medium, and 0.14 large effects.

## RESULTS

### Substrate utilization and energy expenditure during VO_2_max test

**FIG. 2:**
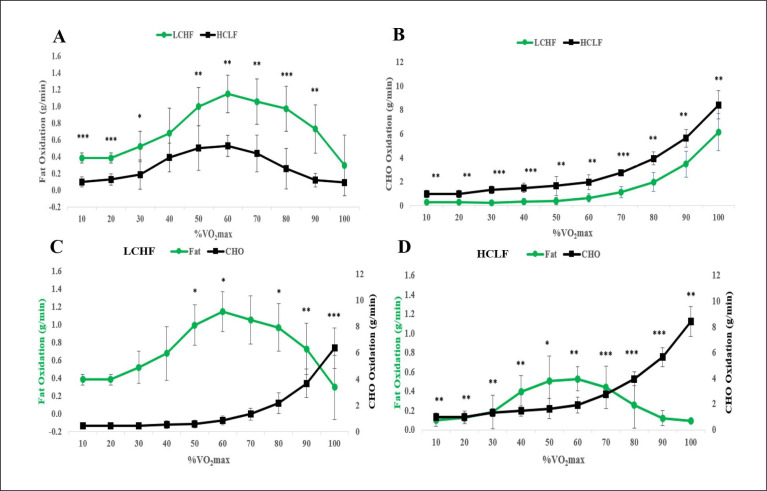
Substrate utilization across a range of exercise intensities during the VO_2_max test in subjects who had eaten the LCHF or HCLF diet for 6 weeks. A, rates of fat oxidation; B, rates of carbohydrate oxidation; C, substrate utilization on LCHF diet; D, substrate utilization on HCLF diet. n = 7. Data: Mean ± SD. *p<0.05, **p<0.01, ***p<0.001, significant difference between LCHF and HCLF.

For rates of fat oxidation, main effects for intensity (p < 0.001, η2p = 0.827), condition (p < 0.001, η2p = 0.936), and interaction effect (p = 0.001, η2p = 0.403) were observed (Figure 2A; Table 2). Subsequently, rates of fat oxidation were higher in 8 out of the 10 stages (excluding, 40 and 100% VO_2_max) in LCHF compared to HCLF (HCLF, 0.28 ± 0.14 g/min; LCHF, 0.72 ± 0.22 g/min; all p’s < 0.015). Figure 2A shows that rates of fat oxidation increased in subjects after both dietary interventions peaking at an exercise intensity of about 60% VO_2_max. However, rates of fat oxidation were significantly higher following the LCHF diet so that even at 90% VO_2_max, rates were substantially higher (0.73 ± 0.29 g/min) than the highest values (0.53 ± 0.13 g/min) achieved at 60% VO_2_max following the HCLF diet. For rates of CHO oxidation, main effects for intensity (p < 0.001, η2p = 0.971), condition (p < 0.001, η2p = 0.932), and an interaction effect (p < 0.001, η2p = 0.592) were observed (Figure 2B; Table 2). Rates of carbohydrate oxidation rose exponentially during progressive exercise in both groups (Figure 2B). Specifically, rates of carbohydrate oxidation were higher at each stage in HCLF (HCLF, 2.91 ± 0.55 g/min; LCHF, 1.49 ± 0.53 g/min; all p’s < 0.004).

**TABLE 2:** Metabolic responses during progressive exercise test to exhaustion

	%VO_2_max										
	10	20	30	40	50	60	70	80	90	100	*P-Value*
**Fat Oxidation (g/min)**											
LCHF	0.38 ± 0.06	0.38 ± 0.06	0.52 ± 0.18	0.68 ± 0.30	1.00 ± 0.23	1.15 ± 0.22	1.06 ± 0.27	0.97 ± 0.27	0.73 ± 0.29	0.30 ± 0.36	Time, *P* < 0.001; η2p = 0.827 Condition, *P* < 0.001; η2p = 0.936 Interaction, *P* = 0.001; η2p = 0.403
HCLF	0.10 ± 0.06	0.13 ± 0.07	0.19 ± 0.17	0.39 ± 0.17	0.50 ± 0.27	0.53 ± 0.13	0.44 ± 0.22	0.26 ± 0.24	0.12 ± 0.08	0.09 ± 0.02	

**CHO Oxidation (g/min)**											
LCHF	0.26 ± 0.15	0.26 ± 0.15	0.24 ± 0.19	0.33 ± 0.29	0.38 ± 0.31	0.64 ± 0.34	1.13 ± 0.47	1.97 ± 0.80	3.48 ± 1.09	6.16 ± 1.56	Time, *P* < 0.001; η2p = 0.971 Condition, *P* < 0.001; η2p = 0.932 Interaction, *P* < 0.001; η2p = 0.592
HCLF	0.97 ± 0.34	0.97 ± 0.34	1.32 ± 0.31	1.48 ± 0.39	1.65 ± 0.79	1.95 ± 0.62	2.76 ± 0.26	3.95 ± 0.55	5.65 ± 0.75	8.43 ± 1.18	

**% Total EE Fat**											
LCHF	78.3 ± 11.1	79.7 ± 12.0	87.6 ± 14.8	90.1 ± 14.3	92.1 ± 11.8	85.1 ± 11.7	72.4 ± 11.6	59.0 ± 12.9	39.0 ± 16.0	12.9 ± 19.8	Time, *P* < 0.001; η2p = 0.926 Condition, *P* < 0.001; η2p = 0.959 Interaction, *P* < 0.001; η2p = 0.555
HCLF	27.3 ± 7.3	25.7 ± 3.0	33.1 ± 14.9	38.9 ± 16.2	43.6 ± 19.8	38.9 ± 10.2	30.1 ± 9.3	16.4 ± 11.9	2.3 ± 6.0	0.0 ± 0.0	

**% Total EE CHO**											
LCHF	21.7 ± 11.1	20.3 ± 12.0	12.4 ± 14.8	9.9 ± 14.3	7.9 ± 11.8	14.9 ± 11.7	27.6 ± 11.6	41.0 ± 12.9	61.0 ± 16	87.1 ± 19.8	Time, *P* < 0.001; η2p = 0.926 Condition, *P* < 0.001; η2p = 0.959 Interaction, *P* < 0.001; η2p = 0.555
HCLF	72.7 ± 7.3	74.3 ± 3.0	66.9 ± 14.9	61.1 ± 16.2	56.4 ± 19.8	61.1 ± 10.2	69.9 ± 9.3	83.6 ± 11.9	97.9 ± 6.0	100 ± 0.0	

Figure 2C compares the rates of fat and carbohydrate oxidation with increasing exercise intensity following the LCHF diet; Figure 2D compares the same measurements following the HCLF diet. For substrate utilization on the LCHF diet, main effects for intensity (p < 0.001, η2p = 0.959), condition (p = 0.013, η2p = 0.667), and interaction effect (p < 0.001, η2p = 0.908) were observed (Figure 2C; Table 2). Specifically, rates of fat oxidation were significantly higher than rates of carbohydrate oxidation at 50 and 60% VO_2_max (p = 0.018 and 0.043, respectively). However, rates of carbohydrate oxidation were significantly higher than rates of fat oxidation at 80, 90, and 100% VO_2_max (p = 0.043, 0.002, and < 0.001, respectively). In contrast, for substrate utilization on the HCLF diet, main effects for intensity (p < 0.001, η2p = 0.978), condition (p < 0.001, η2p = 0.965), and interaction effect (p < 0.001, η2p = 0.971) were observed (Figure 2C; Table 2). Specifically, when on HCLF diet, rates of CHO oxidation were significantly higher than rates of fat oxidation at each exercise intensity (all p’s < 0.05).

**FIG. 3:**
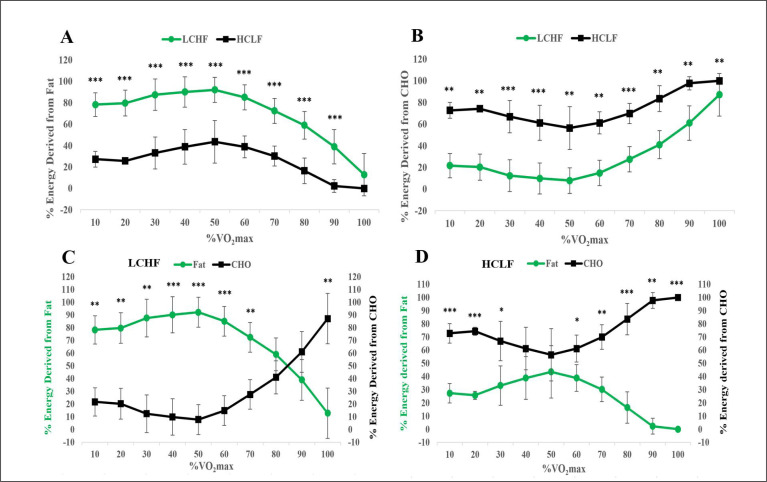
Relative contribution (%) of carbohydrate and fat to energy expenditure during exercise across a range of intensities in response to consuming a LCHF or HCLF diet for 6 weeks. A, energy expenditure from fat oxidation; B, energy expenditure from carbohydrate oxidation; C, energy expenditure on LCHF diet; D, energy expenditure on HCLF diet., n = 7. Data: Mean ± SD. *p<0.05, **p<0.01, ***p<0.001, significant difference between LCHF and HCLF.

Figures 3A and 3B shows the percentage of energy contribution from fat and carbohydrate with increasing exercise intensity following the LCHF and HCLF diets whereas figures 3C and 3D provide a direct comparison of these data. For the relative contribution of fat to energy expenditure, main effects for intensity (p < 0.001, η2p = 0.926), condition (p < 0.001, η2p = 0.959), and an interaction effect (p < 0.001, η2p = 0.555) were observed (Figure 3A; Table 2). The relative contribution of fat to energy expenditure was higher in 9 out of the 10 stages (excluding, 100% VO_2_max) in LCHF compared to HCLF (HCLF, 25.6 ± 2.8%; LCHF, 69.6 ± 4.4%; all p’s < 0.001; Figure 3A). Comparatively, for the relative contribution of carbohydrate to energy expenditure, main effects for intensity (p < 0.001, η2p = 0.926), condition (p < 0.001, η2p = 0.959), and an interaction effect (p < 0.001, η2p = 0.555) were observed (Figure 3B; Table 2). The relative contribution of carbohydrate to energy expenditure was higher at each stage in HCLF compared to HCLF (HCLF, 74.4 ± 2.8%; LCHF, 30.4 ± 4.4%; all p’s < 0.004; Figure 3B).

For energy expenditure (EE) on LCHF diet, main effects for intensity (p < 0.001, η2p = 0.939), condition (p = 0.004, η2p = 0.771), and an interaction effect (p < 0.001, η2p = 0.923) were observed. Specifically, the relative contribution of fat to energy expenditure was significantly higher than carbohydrate at 10–70% VO_2_max (all p’s < 0.003). However, the relative contribution of carbohydrate to energy expenditure was significantly higher than fat at 100% VO_2_max (p = 0.003; Figure 3C, Table 2). In contrast, for energy expenditure on HCLF diet, main effects for intensity (p < 0.001, η2p = 0.939), condition (p < 0.001, η2p = 0.924), and an interaction effect (p < 0.001, η2p = 0.762) were observed. Specifically, when on HCLF diet, relative contribution of carbohydrate to energy expenditure was significantly higher than fat in 8 out of the 10 stages (excluding 40 and 50% VO_2_max; all p’s < 0.029; Figure 3D, Table 2).

### Substrate utilization and energy expenditure during 5-km running time trial

**FIG. 4:**
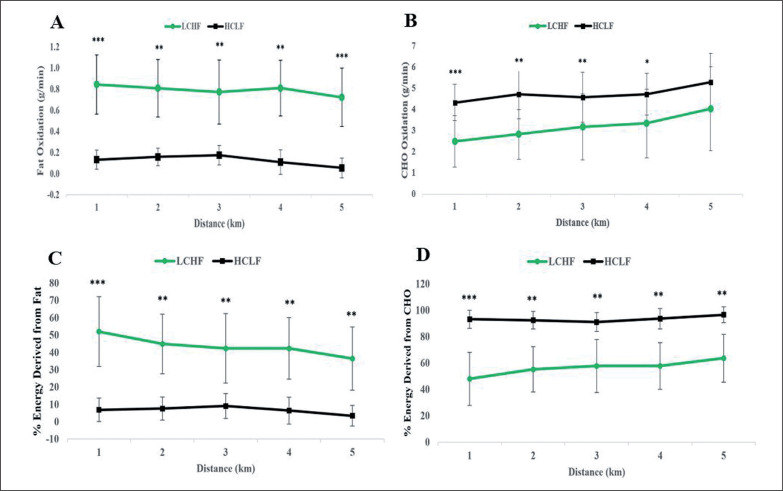
Substrate utilization during 5-km running time trial in response to consuming a LCHF or HCLF diet for 6 weeks. A, rates of fat oxidation; B, rates of carbohydrate oxidation; C, energy expenditure from fat oxidation; D, energy expenditure from carbohydrate oxidation. n = 7. Data: Mean ± SD. *p<0.05, **p<0.01, ***p<0.001, significant difference between LCHF and HCLF.

Figure 4 shows the contribution of fat and carbohydrate oxidation to energy production during the 5KTT after 6 weeks adaptation to either the LCHF or HCLF diets. High rates of fat oxidation and low rates of carbohydrate oxidation were sustained throughout the 5KTT following the LCHF intervention whereas carbohydrate oxidation was substantially higher at all times following the HCLF dietary intervention (Figures 4A and 4B). Specifically, for rates of fat oxidation, a main effect for condition (p < 0.001, η2p = 0.899) was observed (Figure 4A; Table 3). Rates of fat oxidation were higher at each one-km interval in LCHF compared to HCLF (HCLF, 0.13 ± 0.02 g/min; LCHF, 0.79 ± 0.10 g/min; all p’s < 0.002; Figure 4A). For rates of CHO oxidation, main effects for intensity (p = 0.009, η2p = 0.420), and condition (p = 0.003, η2p = 0.795), were observed (Figure 4B; Table 3). Consequently, rates of carbohydrate oxidation were higher in 4 of the 5 one-km intervals (excluding, fifth kilometre) in HCLF (HCLF, 4.73 ± 0.35 g/min; LCHF, 3.18 ± 0.54 g/min; all p’s < 0.022; Figure 4B). For the relative contribution of fat to energy expenditure, a main effect for condition (p = 0.001, η2p = 0.862), and an interaction effect (p = 0.002, η2p = 0.504) was observed (Figure 4C; Table 3). The relative contribution of fat to energy expenditure was higher at each one-km interval in LCHF compared to HCLF (HCLF, 6.7 ± 0.66%; LCHF, 43.6% ± 1.35%; all p’s < 0.004; Figure 4C). Comparatively, for the relative contribution of carbohydrate to energy expenditure, a main effect for condition (p = 0.001, η2p = 0.862), and an interaction effect (p = 0.002, η2p = 0.504) was observed (Figure 4D; Table 3). The relative contribution of carbohydrate to energy expenditure was higher at each one-km interval in HCLF compared to LCHF (HCLF, 93.3 ± 0.66%; LCHF, 56.4 ± 1.35%; all p’s < 0.004; Figure 4C).

**TABLE 3:** Metabolic Responses During 5-km Running Time Trial

	Distance (km)					
	1	2	3	4	5	*P*-Value
**Fat Oxidation (g/min)**						
LCHF	0.84 ± 0.28	0.81 ± 0.27	0.77 ± 0.30	0.81 ± 0.26	0.72 ± 0.27	Time, P = 0.085; η2p = 0.280 Condition, *P <* 0.001; η2p = 0.899 Interaction, *P* = 0.167; η2p = 0.228
HCLF	0.13 ± 0.09	0.16 ± 0.08	0.17 ± 0.09	0.11 ± 0.11	0.05 ± 0.09	

**CHO Oxidation (g/min)**						
LCHF	2.50 ± 1.22	2.82 ± 1.16	3.17 ± 1.55	3.33 ± 1.63	4.04 ± 1.98	Time, P = 0.009; η2p = 0.420 Condition, *P* = 0.003; η2p = 0.795 Interaction, *P* = 0.284; η2p = 0.183
HCLF	4.33 ± 0.84	4.71 ± 1.14	4.59 ± 1.18	4.73 ± 0.97	5.30 ± 1.34	

**% Total EE Fat**						
LCHF	52.0 ± 20.1	44.9 ± 17.2	42.3 ± 17.8	42.3 ± 17.8	36.4 ± 18.2	Time, P = 0.062; η2p = 0.302 Condition, *P* = 0.001; η2p = 0.862 Interaction, *P* = 0.002; η2p = 0.504
HCLF	6.85 ± 6.82	7.57 ± 6.65	9.00 ± 7.18	6.43 ± 7.76	3.43 ± 5.97	

**% Total EE CHO**						
LCHF	48.0 ± 20.1	55.1 ± 17.2	57.7 ± 17.8	57.7 ± 17.8	63.6 ± 18.2	Time, P = 0.062; η2p = 0.302 Condition, *P* = 0.001; η2p = 0.862 Interaction, *P* = 0.002; η2p = 0.504
LCHF	93.1 ± 6.82	92.4 ± 6.65	91.0 ± 7.18	93.6 ± 7.76	96.6 ± 5.97	

## DISCUSSION

The first important finding of this study was that, compared to the HCLF diet, eating the LCHF diet for 42 days significantly altered the metabolic response during both VO_2_max testing and during the 5KTT but without altering physical performance either positively or negatively [47]. This refutes the null hypothesis that we tested. This establishes that the performance in both forms of exercise was not materially influenced by either the pre-exercise diet, or the nature of the metabolic response that these different diets produced during the subsequent exercise tests.

The second important point was the LCHF diet markedly increased fat oxidation and reduced carbohydrate oxidation. Importantly, lower rates of carbohydrate oxidation were not associated with any impairment of performance during either the VO_2_max test or the 5KTT [47]. This contradicts the popular opinion that high rates of carbohydrate oxidation are essential to achieve and to sustain performance during high intensity exercise – the so-called “carbohydrate dependence” of high intensity exercise.

Thus, the rate of fat oxidation at 60% VO_2_max increased two-fold and at 80% VO_2_max more than four-fold (Figure 2A) during VO_2_max testing following the LCHF diet. As a result, fat oxidation provided the majority of energy at all exercise intensities below 85% VO_2_max during the VO_2_max test (Figure 3A); whereas carbohydrate was the predominant fuel at all exercise intensities following the HCLF diet (Figure 3D). Even at 100% VO_2_max, close to 20% of energy was derived from fat oxidation following the LCHF diet.

Figure 3C shows that following the LCHF diet, athletes were able to generate 50% or more of their energy requirements from the oxidation of fat at exercise intensities up to 90% VO_2_max. Consequently, the crossover point for substrate utilization, i.e., the exercise intensity above which CHO oxidation is the predominant source of energy provision [27], occurred at ~85% VO_2_max in athletes eating the LCHF diet (Figure 3C). In contrast following the HCLF diet, carbohydrate provided more than 50% of the total energy consumption at all exercise intensities from rest to 100% VO_2_max (Figure 3D). Thus, for comparison, at 85% VO_2_max, energy use came equally from fats and carbohydrates following the LCHF diet. Whereas at the same exercise intensity following the HCLF diet, 90% of the energy provision came from carbohydrate oxidation with only 10% from fat oxidation. Similarly, during the 5KTT, ~56% of energy was derived from fat oxidation following the LCHF diet (Figure 4C) whereas more than 93% of the energy came from carbohydrate oxidation following the HCLF diet (Figure 4D).

These data therefore show that the “carbohydrate dependence” of higher intensity exercise is an artifact of the habitual diet eaten in the days and weeks before the exercise bout. However, this “carbohydrate dependence” can be readily modified with the adoption of a high-fat diet. This is not a novel finding, having been repeatedly shown in the past [42, 43] and more recently [24, 44–46].

The third important finding is that when adapted to the LCHF diet, subjects showed enhanced, not impaired metabolic flexibility since they retained the capacity to use carbohydrates at high rates at 100% VO_2_max (Figure 2B; Figure 2C); yet they were able to provide more than 50% of their energy from fat oxidation even at an exercise intensity of 85% VO_2_max (figure 2C).

In contrast, following the HCLF diet, during exercise at any intensity above 50% VO_2_max, carbohydrate-adapted subjects could increase their energy expenditure only by increasing rates of carbohydrate oxidation (Figure 3D) making their metabolism “carbohydrate dependent” at all exercise intensities above 50% VO_2_max. But when adapted to the LCHF diet, subjects were able to extract most of their energy from the use of fat at all exercise intensities below 85% VO_2_max, with the result that their metabolism became “carbohydrate dependent” only at exercise intensities > 85% VO_2_max.

Indeed, one of the potentially misleading features of Figure 1 is to imply that fat oxidation begins to fall precipitously at all exercise intensities above ~65% VO_2_max, and essentially ceases during exercise at 85% VO_2_max or higher. Rather our data show that there is no sudden precipitous decline in fat oxidation with increasing exercise intensity in athletes adapted to the LCHF diet. Instead, even at ~85% VO_2_max, fat oxidation continues to contribute a proportion of energy equal to that of carbohydrate oxidation (Figure 3C). However, in agreement with the finding of Achten and Jeukendrup [22], the contribution of fat oxidation to total energy expenditure in carbohydrate-adapted athletes, begins to fall at exercise intensities above 60%, reaching zero at 90% VO_2_max (Figure 3D).

Thus, the LCHF diet increases metabolic flexibility by allowing higher rates of fat oxidation whilst retaining the capacity to exponentially increase carbohydrate oxidation rates at exercise intensities above 70% VO_2_max. Even then, high rates of fat oxidation are still maintained at those high exercise intensities. In contrast following the HCLF diet, carbohydrates become the predominant source of energy at all exercise intensities above 70% VO_2_max.

In addition to the study being limited by a small sample size an important limitation of the metabolic data from the VO_2_max test is that those data were not collected during steady state exercise. As a result, the respiratory exchange ratio (RER) used to calculate the relative contributions of fat and carbohydrate oxidation to total energy expenditure, will be different from values measured during more prolonged steady state exercise, more especially at higher exercise intensities. For example, when exercise intensity exceeds an individual’s maximal lactate steady state, shifts in acid-base balance occurs. During increased glycolytic flux, lactate accumulation in the contracting muscle moves to the extracellular fluid and increases [H^+^], which is buffered by [HCO3^-^], thus increasing the production of CO_2_ and elevating the VCO_2_. As a result, indirect calorimetry overestimates carbohydrate oxidation and underestimates fat oxidation during high intensity exercise [51]. But in the context of this experiment, the net effect would have been to under- and not to over-estimate the contribution of fat oxidation to overall energy balance at higher exercise intensities following both dietary conditions. Thus, this limitation would increase, not reduce, the validity of the arguments that we present.

## CONCLUSIONS

In summary our previous study [47] found that performance during either VO_2_max testing or during a 5KTT was unaffected by dietary adaptation to a LCHF diet. In this study we show that the dietary change produces a dramatic alteration in the contribution of carbohydrate or fat metabolism to energy production during both forms of exercise. Importantly the major change was to increase the proportion of energy derived from fat oxidation even at high exercise intensities.

Accordingly, the study effectively challenges three currently popular doctrines. First, the belief that a high-fat diet impairs metabolic flexibility during exercise. In contrast, we show that metabolic flexibility is increased by the LCHF diet whereas it is compromised by the HCLF diet. Second, the belief that exercise of high intensity is “carbohydrate dependent”. Instead, we show that this “carbohydrate dependence” of high intensity exercise is an artefact of the athlete’s preceding diet. Third, the belief that because of this “carbohydrate dependence” of high intensity exercise, fat cannot substitute for carbohydrate use during high intensity exercise without impairing exercise performance. Instead, we show that adaptation to the LCHF and HCLF diets produce matching exercise performances. Which might suggest that dietary macronutrient composition may have a much smaller effect on human exercise performance than is currently believed [52].
